# Optimisation of an Electrochemical DNA Sensor for Measuring KRAS G12D and G13D Point Mutations in Different Tumour Types †

**DOI:** 10.3390/bios11020042

**Published:** 2021-02-05

**Authors:** Bukola Attoye, Matthew J. Baker, Fiona Thomson, Chantevy Pou, Damion K. Corrigan

**Affiliations:** 1 Department of Biomedical Engineering, University of Strathclyde, 40 George Street, Glasgow G1 1QE, UK; damion.corrigan@strath.ac.uk; 2 Technology and Innovation Centre, Department of Pure and Applied Chemistry, University of Strathclyde, 99 George Street, Glasgow G1 1RD, UK; matthew.baker@strath.ac.uk; 3 Wolfson Wohl Cancer Research Centre, Institute of Cancer Sciences, University of Glasgow, Glasgow G61 1QH, UK; fiona.thomson@glasgow.ac.uk (F.T.); chantevy.pou@glasgow.ac.uk (C.P.)

**Keywords:** electrochemical, DNA biosensors, KRAS, liquid biopsy, cancer point-of-care diagnostic tests

## Abstract

Circulating tumour DNA (ctDNA) is widely used in liquid biopsies due to having a presence in the blood that is typically in proportion to the stage of the cancer and because it may present a quick and practical method of capturing tumour heterogeneity. This paper outlines a simple electrochemical technique adapted towards point-of-care cancer detection and treatment monitoring from biofluids using a label-free detection strategy. The mutations used for analysis were the KRAS G12D and G13D mutations, which are both important in the initiation, progression and drug resistance of many human cancers, leading to a high mortality rate. A low-cost DNA sensor was developed to specifically investigate these common circulating tumour markers. Initially, we report on some developments made in carbon surface pre-treatment and the electrochemical detection scheme which ensure the most sensitive measurement technique is employed. Following pre-treatment of the sensor to ensure homogeneity, DNA probes developed specifically for detection of the KRAS G12D and G13D mutations were immobilized onto low-cost screen printed carbon electrodes using diazonium chemistry and 1-ethyl-3-(3-dimethylaminopropyl) carbodiimide hydrochloride/N-hydroxysuccinimide coupling. Prior to electrochemical detection, the sensor was functionalised with target DNA amplified by standard and specialist PCR methodologies (6.3% increase). Assay development steps and DNA detection experiments were performed using standard voltammetry techniques. Sensitivity (as low as 0.58 ng/μL) and specificity (>300%) was achieved by detecting mutant KRAS G13D PCR amplicons against a background of wild-type KRAS DNA from the representative cancer sample and our findings give rise to the basis of a simple and very low-cost system for measuring ctDNA biomarkers in patient samples. The current time to receive results from the system was 3.5 h with appreciable scope for optimisation, thus far comparing favourably to the UK National Health Service biopsy service where patients can wait for weeks for biopsy results.

## 1. Introduction

In recent years, analytical electrochemistry has emerged as a powerful tool for the rapid in-vitro analysis of biological analytes for the early detection of certain diseases, such as cancer [1]. Cancer is a genetic disease by nature, caused by mutations in certain genes thereby resulting in cellular malfunction [2]. Imaging tests can sometimes be inconclusive and broadly do not provide information on the stage or type of cancer, so further biopsy is needed [3]. Serious medical risks and related metastasis may ensue from gathering multiple biopsies from different regions of a primary tumour [4].

The period at which a tumour shows clinical symptoms usually corresponds with the later stages of progression (e.g., Phases III and IV), when the cancer is metastatic or unresectable, causing surgery and therapy to be less effective. In addition, surgical biopsy procedures are not possible or recommended for some patients; therefore, liquid biopsies that are able to detect the presence of tumour DNA hold promise as a non-invasive alternative.

**Figure 1:**
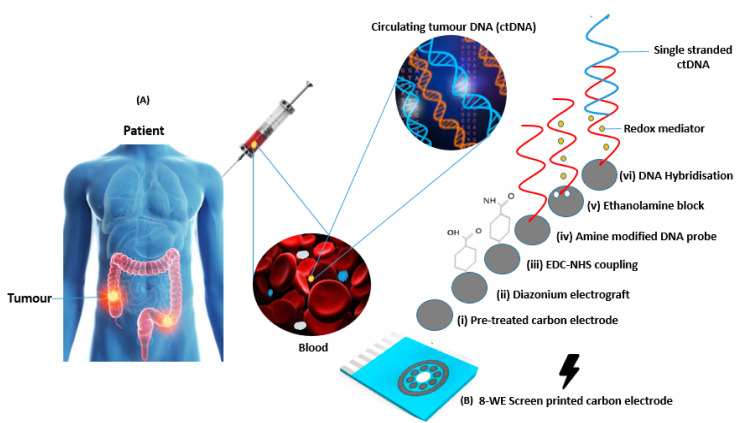
(**A**) Schematic showing circulating tumour DNA (ctDNA) retrieval and analysis [33,34,35] (**B**) Image of a screen-printed electrode array employing eight working electrodes with a common Ag reference and carbon counter electrodes along with a schematic showing modification steps and DNA functionalisation.

Most body fluids, including blood, contain tumour biomarkers and short fragments of cell-free DNA (cfDNA) that can be detectable as shown in Figure 1A below. In cancer patients, a fraction of cfDNA called circulating tumour DNA (ctDNA) can be found, which emerges from tumours and may feature the same mutations and genetic modifications as those present in the primary tumour [5]. While circulating tumour cells (CTCs) that have been shed into the vasculature of a primary tumour are also transported around the body in circulation, they are present at quantities of around 10 cells/mL of blood, suggesting that only very low concentrations are present in clinical samples. In contrast to ctDNA, CTCs are rare in peripheral blood and are difficult to separate from other cells, increasing the credibility for the use of ctDNA in liquid biopsy applications. The mechanism of ctDNA release from tumour cells is poorly understood [6], however it is thought to be released in small quantities following apoptosis or necrosis. ctDNA typically comprises 0.01–1% of the circulating free DNA in blood [7] and it is important to note that this can be shed as both double stranded and single stranded DNA [8]. At present, ctDNA can be detected in blood and other body fluids like lymph, urine and stool [9]. Due to the small fraction of ctDNA concealed by large background levels of wild-type cell-free DNA, sensitive amplification reactions such as polymerase chain reaction (PCR) will need to be implemented to achieve detection and discrimination above wild type signals. A point of care (PoC) measurement of circulating tumour DNA (ctDNA) may offer a non-invasive strategy for evaluating response to treatment, monitoring disease recurrence, capturing tumour heterogeneity and gaining insights into a tumour’s mutational profile [9,10].

Single nucleotide variations (SNV) in the Kirsten rat sarcoma viral oncogene homolog, commonly abbreviated ‘KRAS’ are present across many human tumour types with KRAS G12D and G13D being specific variants observed. KRAS is a member of the RAS family of proteins which are a part of at least six signalling pathways in a healthy human cell and is the most commonly mutated protein across many human tumour types [11]. KRAS mutations take place in approximately 90% of pancreatic cancers [12], 30% of lung cancers [13], 60% of thyroid cancers and 43% of colorectal cancers [14]. KRAS activated mutations drive cancer initiation, progression and drug resistance, directly leading to nearly a million deaths per year. SNVs have been used as biomarkers for predicting disease risk [15,16], and its combination with liquid biopsies will create innovations in biomarker detection that will enhance clinical outcomes for patients at all cancer stages [17].

From a PoC viewpoint, Electrochemical DNA biosensors represent an exciting approach in the detection of clinically important biomarkers due to their rapidity and simplicity [18,19,20]. Electrochemical biosensors are used to directly convert a biological binding event to an electronic signal [21]. A range of electrode materials and electrochemical measurement approaches have been employed for sensitive measurements [8,22,23,24]. The possibilities of electrochemical biosensors, once matured as a technology to provide efficient clinical workflows, is vast. In electrochemical DNA biosensing, a change in signal is obtained when recognition and hybridisation of two opposing strands of DNA occur as a result of their base-pair complementarity. A double stranded DNA sequence with tumour-specific mutations can indicate the diagnosis of a specific cancer [24]. As the concentration of ctDNA is directly proportional to the tumour grade, attaining high sensitivity for the DNA sensor is key for the early detection of disease, developing tailored therapies and monitoring therapy efficiency.

Given the continued need for the miniaturization of advanced electronics, the area of screen printing techniques has been adapted for electronic circuit fabrication. Screen printed electrodes (SPEs) are evolving as they are easy to use and can be produced on a large scale. SPEs are also very practical as they are disposable and low cost when manufactured in large volumes. SPEs are usually composed of working electrodes made of conductive inks like carbon, platinum, gold or silver. Although, carbon with organic solvents, binding pastes and some additives that provide functional characteristics are contained in conductive inks found in Screen Printed Carbon electrodes (SPCEs), they can be modified in order for their electrochemical properties to be improved [24]. Carbon electrodes are also chemically inert, specifically at negative potential ranges in all media, making them particularly suitable electrode sensors for electroanalytical chemistry, providing an advantage over metal electrodes [25]. SPCEs are simple, sensitive, cost-effective (~£2 each) and disposable, making them preferable for rapid electrochemical analyses and suitable as electrodes for characterizing the processes implemented herein, specifically for the detection of ctDNA.

The SPCE sensor shown in this study was developed by characterising the surface of the electrode chip to determine the treatments and buffers with optimal sensitivity. In order to make the surface of the SPCE as homogenous as possible, it is important that they are pre-treated. These pre-treatments remove any binder residues left on the carbon surface after the curing process [26,27,28,29], with well-established electrochemical oxidative pre-treatments not only showing removal of binder residues left on the surface of SPCE after curing but also improvement of carbon surface sensitivity [30]. In this study, two common buffers are compared for pre-treatment: NaOH and NaCl. Up until now, few studies have been done on electrochemically pre-treating and characterising the surface of activated screen printed carbon electrodes [28,29,30]. We make the choice of a characterisation redox buffer after observing the effect of surface chemistry in relation to electron transfer rates using an inner-sphere redox mediator (Ferri-ferrocyanide) and an outer-sphere redox mediator (ruthenium hexaminechloride). Further voltammetric characterisation is performed to reveal DNA hybridisation effects and thus mutation detection in both potassium ferri-ferrocyanide (1 mM Fe(CN)_6_^3−/4−^ in 0.1× PBS) and ruthenium hexaminechloride (1 mM Ru(NH_3_)_6_Cl_3_ in 0.1× PBS) solutions.

This work presents a KRAS G12D and G13D DNA oligonucleotide probe modified sensor array that can accurately detect mutant KRAS amplicons and therefore forms the basis of a system for the accurate detection of ctDNA in patient samples and monitoring of response during treatment. This was achieved by amplifying mutant DNA isolated from a human cancer cell line recovered from clinical samples, using electrochemical techniques and SPCEs to detect a clinically relevant mutation, comparing the signal change from DNA hybridisation experiments involving amplified KRAS mutant samples and amplified wild-type KRAS samples, varying concentration of amplified products to determine concentration effects and establishing a limit of detection for the DNA amplification reaction. Cyclic Voltammetry (CV), Square Wave Voltammetry (SWV) and Differential Pulse Voltammetry (DPV) are routinely used electrochemical measurement techniques that supply information on electron transfer reaction kinetics of any combined chemical reaction [31]. In these techniques, a potential waveform is applied to the working electrode (WE). The peak current obtained is directly influenced by hybridisation between target and immobilised probe DNA strands [32]. In this study, DPV, SWV and CV were used depending on whether electrodes needed to be cleaned, electrografted, or characterised during sensor measurement. Considering the choice of steps and ease of use of the assay being developed, the system can be very easily automated and integrated into a final device capable of fast and seamless clinical measurements. The presented work builds on a recent publication [1] showing the possible detection of KRAS G12D mutations, by developing understanding or surface pre-treatment steps (essential to realising a reproducible analytical technique) and by introducing the detection of the KRAS G13D mutation which expands the assay towards a multi-marker assay and permits the analysis of more tumour types.

## 2. Materials and Methods

### 2.1. Reagents

Supermix for probes, Droplet digital PCR (ddPCR) assays, DG8TM cartridges and Droplet Generation Oil were obtained from Bio-Rad Laboratories Ltd., Hertfordshire, UK. Deionized water, sodium chloride, sodium hydroxide, phosphate-buffered saline (PBS), sodium nitrate, 4-aminobenzoic acid, hydrochloric acid, ethanolamine, 2-(N-morpholino) ethanesulfonic acid (MES), 1-ethyl-3-(3-dimethylaminopropyl) carbodiimide hydrochloride (EDC), N-hydroxysuccinimide (NHS), hexammineruthenium (III) chloride, potassium ferricyanide, potassium chloride and potassium ferrocyanide were all purchased from Sigma–Aldrich, (Dorset, UK). Two hundred and fifty units of HotStarTaq Plus and dNTP Mix, PCR Grade (200 μL), were purchased from Qiagen, (Manchester, UK). Phusion Direct PCR kit was purchased from thermo fisher scientific (Renfrew, UK).

### 2.2. Sensor Development and Set-Up

A multiplex system comprising screen-printed multi-carbon electrodes (DRP 8W110), a potentiostat, a multiplexer and a connector were set up for electrochemical measurements. The chip containing eight carbon working electrodes with diameters of 2.95 mm each, a carbon counter and silver reference electrode as shown in Figure 1B above were obtained from DropSens (Oviedo, Spain) with chip dimensions of 50 × 27 × 1 mm (L × W × D). The screen-printed fabrication process was specified by the manufacturers.

### 2.3. Electrode Preparation and Surface Functionalisation

All electrochemical measurements were recorded using PS-Trace software. DNA hybridisation experiments were performed using a covalently attached layer of single-stranded DNA probes. The surface functionalisation protocol is illustrated in Figure 1B. To prepare the surface of the carbon electrodes for DNA probe attachment, it was necessary to first use a surface pre-treatment method by applying 1.4 V for 1 min in 0.5 M acetate buffer solution (ABS) containing 20 mM NaCl 8 (pH 4.8) via CV. An alternative pre-treatment technique explored for optimisation comparison required soaking the SPCEs in 3 M NaOH for 1 h as an initial step and anodizing at 1.2 V using a scan rate of 0.5 V/s via CV. Next, 2 mM NaNO_2_ solution with 2 mM 4-aminobenzoic acid was prepared in 0.5 M HCl and stirred for approximately 5 min at room temperature to produce a diazonium compound. The activated diazonium solution was then scanned using CV from +0.4 to −0.6 V at a scan rate of 100 mV/s followed by a wash with deionised (DI) water. The resulting 4-carboxyphenyl (AP) film was activated on the electrode’s surface with 100 mM EDC and 20 mM NHS in 100 mM MES buffer (pH 5.0) for 60 min to form an ester that allowed for efficient conjugation to the amine-modified ssDNA probe. A 1 mM ruthenium and 1 mM potassium ferri-ferrocynide buffer were compared to analyse electron transfer rates. Ferricyanide buffer (5 mM) was used to characterise the sensor surface for DNA detection. All the reported steps and measurements were carried out at room temperature, unless otherwise stated.

### 2.4. Genomic DNA Sample Preparation, DNA Probe Design, and Sample Amplification

Copies of the KRAS pG12D and pG13D mutant and wild-type DNA were amplified from genomic DNA (gDNA) isolated from SK-UT-1 cells (pG12D) and HCT116 cells (pG13D). Levels of both mutant and wild-type DNA were determined using ddPCR assays in combination with a QX200TM Droplet DigitalTM PCR system (Bio-Rad Laboratories Ltd., Hertfordshire, UK) following the manufacturer’s instructions. Briefly, 5–10 ng of gDNA isolated from SK-UT-1 cells was combined with ddPCR Supermix for probes (No dUTP) and fluorescein amidite (FAM)-labelled KRAS p.G12D (KRAS p.G12D c.35G>A, Human (dHsaMDV2510596)) or KRAS p.G13D (KRAS p.G13D c.35G>A, Human (dHsaMDV2510598)) primers/probe and hexachloro-fluorescein 9 (HEX)-labelled KRAS WT primers/probes (KRAS WT for p.G12D c.35G>A and KRAS WT for p.G13D c.38G>A) in the presence of HaeIII restriction enzyme and in a volume of 20 μL. Reaction samples were loaded onto a DG8TM cartridge with 70 μL of droplet generation oil for Probes according to the Droplet Generator Instruction Manual (Bio-Rad Laboratories Ltd., Hertfordshire, UK). The PCR cycling conditions for the generated droplets were as follows: initial enzyme activation at 95 °C for 10 min, followed by 40 cycles of denaturation at 94 °C for 30 s, and annealing/extension at 55 °C for 1 min, after which it ended with a final enzyme deactivation at 98 °C for 10 min. Data acquisition after thermal cycling was performed using the QX200 Droplet Reader and the QuantaSoft Software (Bio-Rad Laboratories Ltd., Hertfordshire, UK).

The PCR primers and probes designed in this study were based on the published sequence of KRAS pG12D and pG13D under accession number NC_000012.12 [36]. Amine-modified synthetic oligonucleotides (KRAS G12D and KRAS G13D) were designed for use as probes, as shown in Table 1 below, with a concentration of 200 μM obtained from Sigma–Aldrich, UK, and stored at −80 °C prior to aliquoting for use as probes. A wild-type probe (without the single base mutation) was also designed for use as a negative control. The DNA probe stocks were diluted to a concentration of 2 μM in 0.1× PBS prior to immobilisation. Primer-BLAST software was used to design the PCR primers used in this study. The forward primers and reverse primers had an estimated GC content of 40–55%, an estimated product length of 88 with low self-complementarity.

Further amplification of extracted wild-type, KRAS G12D and G13D-mutated DNA samples was carried out using the Phusion Direct PCR kit following the protocol and reaction set-up guide outlined by Thermo Scientific, UK. Phusion Blood II DNA polymerase (1 µL), 2× PCR Buffer (25 µL), 50 mM EDTA (2.5 µL), 50 mM MgCl_2_ solution (1.5 µL) and 100% DMSO (2.5 µL) were all included in the reaction mix and dispensed into appropriate PCR tubes. 2.5 μL of Template DNA containing 18.4 ng/μL double stranded DNA, 5 μL forward and reverse primers and 10 μL ultrapure water were added to the master PCR tube containing the reaction mix, and the thermal cycler was programmed to start with an initial heat-activation step at 98 °C for 300 s. Temperature specifications for denaturing, annealing and extending were set at 90 s for 94 °C, 65 °C and 72 °C, respectively. A final extension for 60 s at 72 °C was set, and the PCR conditions were set for 37 cycles. The PCR amplification of wild-type and KRAS G13D samples was performed using the miniPCR thermal cycler [37], and amplicon yields of 117 ng/μL were confirmed using the Qubit 4 fluorometer and dsDNA broad range quantification assay [38].

## 3. Results and Discussion

### 3.1. Assay Workflow and Development

**Figure 2:**
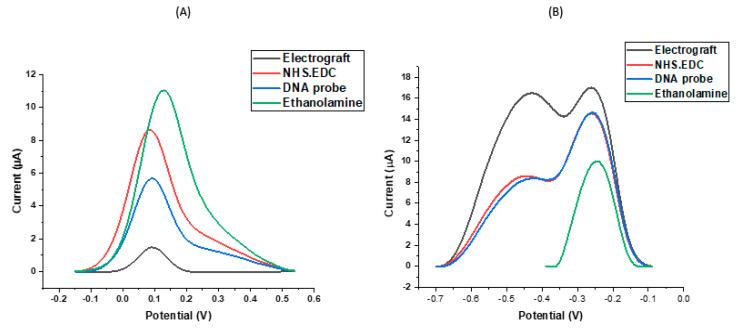
Examines the effect of two different redox agents on the DPV peak current after each functionalisation step on SPCE sensor response characterised using (**A**) 1 mM ferri-ferrocyanide buffer in 0.1× PBS and (**B**) 1 mM ruthenium hexaminechloride in 0.1× PBS.

The use of an SPCE with multiple working electrodes allows each electrode to be individually modified and rapidly carry out simultaneous measurements of peak currents. Electrografting using in situ generated diazonium cations is important for modifying the surface of the SPCE by allowing the formation of covalent bonds between the carbon surface and organic films [39,40,41]. The EDC molecule is an established zero-length cross-linking agent that has been employed in coupling carboxyl groups to primary amines in various applications [41]. One of the main benefits of EDC coupling is its water solubility that allows direct bioconjugation without prior organic solvent dissolution. To improve the stability of the active ester, NHS was introduced to modify the amine-reactive chemical substance by converting it to an active NHS ester, thus maximising the efficiency of the EDC-mediated coupling reactions. The reproducibility of this hybridisation sensor was explored by simultaneously analysing all eight WEs from the multi-electrode chip after pre-treatment and electrochemical signal changes of similar amplitude, direction and magnitude were observed. Figure 2A below shows the effect of each modification step on DPV peak current using ferri-ferrocyanide. The low peak current observed after diazonium reduction can be attributed to the thickness of the film resulting from the covalent bonds created on the surface of the electrode. In Figure 2B, this effect is reversed and a higher peak current with two peaks are noted, suggesting a high sensitivity to the organic films. From the ferri-ferrocyanide characterisation, the NHS-EDC peak reflects both coupling initiation and activation on the surface of the electrode which results in an enhanced oxidation due to a neutrally charged NHS ester, leading to a negative potential shift and an increase in peak current. DNA is negatively charged and thus resulted in a decrease in peak current when immobilised on the sensor surface. There is also an electrostatic repulsion between negative ferri-ferrocyanide ions and the negative phosphate group in the DNA structure. The double peaks from the ruthenium hexaminechloride characterisation make it more difficult to identify the correct peak current. In this case, we attribute the right hand peak to the ruthenium hexamine chloride redox reaction from free solution and the smaller smeared out left hand peak to the ruthenium redox reactions taking place at higher potentials because the redox reporter is trapped in organic layers and electrostatically associating with the DNA strands on the probe modified electrode surface. The multiplexed analysis we used greatly reduced the analysis time because of the high throughput of samples and minimised reagent consumption. After introducing the probe solution to the surface of the modified electrodes, the remaining active groups on the electrode were blocked using ethanolamine to produce a consistent sensing layer in order to facilitate DNA specificity and stability in terms of the DNA binding response. Blocking the free surface on the electrode resulted in an increase in peak current in ferri-ferrocyanide characterisation. A single but decreased current peak is shown in Figure 2B after blocking and this can be attributed to the sensitivity of ruthenium hexaminechloride to the consistent layer on the outer surface of the electrode. The findings from these modification characterisations are in line with previous studies [42,43,44,45] and noting the observations on SPCEs, our optimal characterisation buffer for these studies is ferri-ferrocyanide.

**Figure 3:**
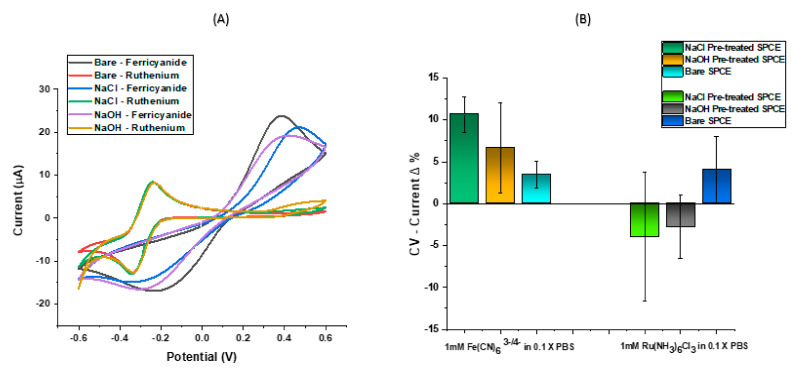
CV measurements showing the effect of an inner sphere and outer sphere redox mediator on electron transfer for different SPCE surfaces on (**A**) non-modified surface and (**B**) modified and DNA functionalised surfaces.

A growing demand for reliable detection devices motivates much biosensor development [44], so it is therefore necessary to improve assay reproducibility and one crucial aspect of this is surface pre-treatment. A high level of consistency in the peak current, potential and width was observed after repeated cycling of each bare electrode, however optimisation work was carried out to ensure that our assay was as sensitive as possible. In Figure 3, it was noted that although the bare SPCE exhibited an admirable sensitivity with the highest peak current of those presented (Figure 3A), after all surface modification steps and DNA hybridisation was carried out, the SPCE pre-treated using acetate buffer containing NaCl showed the most suitable response. As previous studies have shown an increase in surface roughness after pre-treating screen printed electrodes [29,30], we can infer that the improved electrochemical performance after DNA hybridisation on pre-treated electrodes resulted from the ability of the target DNA strands to bind readily to the surface of the probe modified electrode. A high percentage signal change directly implies that a significant reduction in peak current upon target hybridisation has occurred. For a ferri-ferrocyanide characterisation buffer, this can be attributed to the repulsion between negative charges of target DNA, probe DNA and negative ferri-ferrocyanide ions. A wider peak separation (∆E_p_) than that predicted by the Nernst equation is also observed in SPCEs characterised by ferri-ferrocyanide (due to surface effects). Both redox buffers exhibited acceptable reversibility on pre-modified SPCEs. From Figure 3B, the changes in buffer characterisation observed on the DNA modified SPCEs confirms ruthenium hexaminechloride is an outer-sphere electron transfer mediator that is only affected by changes in the electroactive area, while the redox couple ferri-ferrocyanide is useful for determining the existence of functional groups due to its inner sphere sensitivity. The ferri-ferrocyanide buffer gave the most consistent signal change upon DNA hybridisation on the modified SPCEs, particularly when pre-treated with NaCl, therefore giving us a system which could be taken through to a full assay development stage. Upon hybridisation, NaOH pre-treatment indicated a lower sensitivity and a higher variation of sensor surface, thus raising doubts on the suitability of NaOH treatment for SPCEs in DNA hybridisation work.

### 3.2. DNA Sensor Hybridisation Specificity

**Figure 4:**
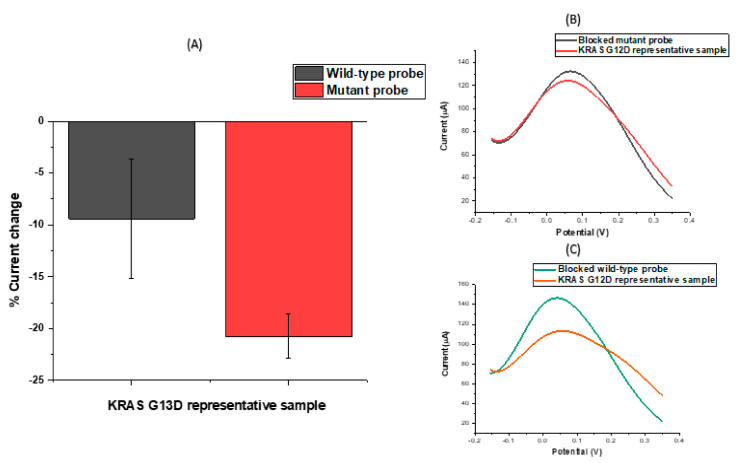
(**A**) CV percentage signal change in response to mutant and wild-type probes hybridized with genomic KRAS G13D ssDNA. (**B**,**C**) DPV signal changes in response to incubated KRAS G12D ssDNA hybridised with mutant and wild-type (WT) oligonucleotides, respectively.

After observing consistent behaviour of modified electrodes on the same chip, the next step was to test the assay’s response to incubation in a representative KRAS sample using the designed probes. We explored the ability of the probe-modified electrodes to discriminate between G13D mutant and wild-type KRAS sequences in representative samples. To investigate specificity levels and gain an initial impression of assay sensitivity, a series of electrodes were functionalised with KRAS G13D mutant and wild-type probe sequences. The results of these experiments are summarised in Figure 4A, which shows the percentage change in the CV peak current following target hybridisation. For macroscale electrodes functionalised with biological molecules, such as DNA or antibodies, the expectation is that differential pulse voltametric peak currents will reduce upon target hybridisation. It has been observed that these effects can be reversed when micro- or nanoscale electrodes are employed [20,46], but for this study, the electrodes used were comfortably on the macro scale (diameter = 2.95 mm). For nanomolar (>10 nM) and micromolar concentrations, an increase in the peak current following hybridisation was consistently observed (and has also been observed in other data from our lab involving SPCEs) [1] for carbon electrodes which is likely explained by the high surface density of hybridised DNA amplicons changing the interfacial properties of the electrode and, therefore, altering the electrochemical response. The underlying physical mechanism of this effect is actively under investigation. Figure 4A shows that when mutant and wild-type oligonucleotide probe sequences functionalised SPCEs were incubated in a representative sample containing the G13D mutation, there was hybridisation in both cases; the signal change was greater for the wild-type probe because of the high background of wild-type DNA and the comparatively low fraction of mutated KRAS G13D present in the representative sample. Similar behaviour was observed for KRAS G12D probe functionalised electrodes for a representative sample for that particular mutation, showing the wild-type KRAS DNA hybridised strongly to the nucleic acid modified carbon surfaces. As a result of these findings and the inability to electrochemically discriminate between positive and negative samples owing to the strong influence of background DNA in the sample, DNA amplification strategies were developed and tested in order to ensure the production of unequivocal detection of ctDNA mutations.

### 3.3. KRAS G13D Amplification and Negative Control

**Figure 5:**
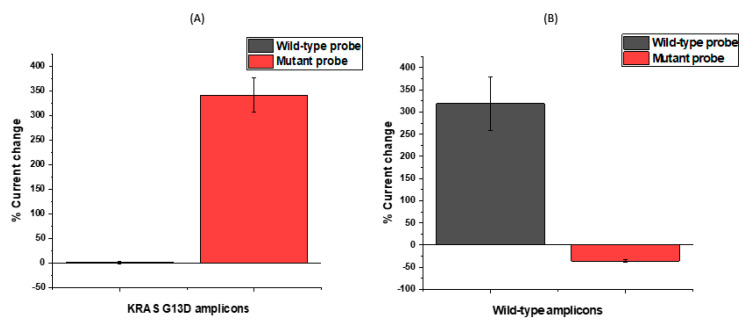
Successful amplification of (**A**) KRAS G13D mutants further confirmed by a large percentage signal change ratio between mutant probe and G13D mutant amplicons (**B**) KRAS wild-type further confirmed by a large percentage signal ratio between wild-type probe and wild-type DNA.

In order to selectively amplify the mutant target from a pool of mutant and wild-type sequences in a sample, primers used for the PCR amplification were tailored by varying the single nucleotide responsible for the mutation in the primer sequence. Adopting this approach allowed us to effectively enrich the number of mutated DNA sequences in the sample without amplifying the wild-type in order to produce a signal change above the background signal generated by the KRAS wild-type DNA non-specifically associating with the oligonucleotide probe sequences for KRAS G13D. In selecting the approach reported here, ctDNA detection could potentially be coupled to a DNA amplification reaction, because it allows the possibility of developing a multiplexed panel of DNA sequences on a single chip, meaning that commonly mutated genes could all be identified in parallel (e.g., KRAS, TP53, BRCA1/2, IDH-1). This concept of developing biomarker “panels” is thought to be one of the key advantages of this approach [47]. From Figure 5A, when the wild-type probe-sequence modified electrodes were hybridised with KRAS G13D amplicons, alterations in the peak current were not observed, indicating no significant hybridisation. The mutant amplicons when incubated with mutant probe modified electrodes gave rise to a very significant signal change (~350%), indicating hybridisation with ultra-concentrated DNA samples (nano–micromolar concentration ranges) because of the strong positive signal change. An opposite response is noted in Figure 5B where wild-type amplicons resulting from DNA amplification using wild type primers are hybridised using wild-type probe modified electrodes. In this case, the wild-type hybridisation exhibited a significant signal change while mutant probe modified electrodes showed no significant hybridisation, representing an additional control. These findings were thoroughly satisfying, i.e., that the surface-tethered KRAS G13D mutant probe sequence could, in fact, discriminate between the mutant and wild-type samples based on the presence or absence of PCR amplicons for KRAS G13D with high sensitivity. This in fact represented a type of double specificity for the PCR-based assay, because the primer design had already been shown to specifically amplify the mutated sequence so coupling in the specificity of the electrochemical probe sequence meant that the assay would be able to successfully discriminate mutant amplicons from the sample. Having established the specificity of the assay and the nature of the electrochemical change, the next step involved verifying the sensitivity of the assay and dose–response effects for the KRAS G13D mutant PCR product.

### 3.4. Concentration Dose Response

**Figure 6:**
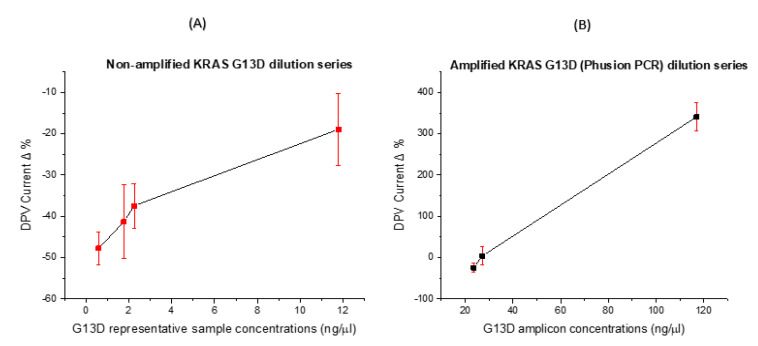
ctDNA response at different concentrations in a dilution series (**A**) DPV Peak currents percentage change for genomic KRAS G13D representative clinical sample at different concentrations (**B**) DPV Peak currents percentage change for KRAS G13D amplicons at different concentrations.

After establishing PCR primer specificity, ssDNA probe specificity and electrochemical signal changes in the correct direction and magnitude, it was important to investigate dose–response effects. In these experiments, non-amplified and amplified samples were diluted and a dose-response curve was constructed (see Figure 6A,B). We expected a reduction in the peak current when specific DNA hybridisation had taken place, and this was found to be the case for lower concentrations of DNA (pico-to-low nanomolar concentrations). For the unamplified sample (Figure 6A), the lowest concentration (0.85 ng/µL) demonstrated the lowest reduction in oxidative peak current post-hybridisation with signal change increasing as sample concentration increased. The problem here, however, was the specificity of the probe–target interaction (as shown earlier) and the relatively small signal change brought about by incubation with unamplified samples. The signal changes were negative, due to the fact that these were relatively low concentrations of DNA, leading to limited hybridisation. On the other hand, the amplified sample produced a dose–response curve with higher signal changes which were positive in direction due to the specific enrichment of the mutant sequence concentration with smaller standard deviations because of the hybridisation of strands with high complementarity (the unamplified samples contained fewer point mutations) and, in effect, the full fraction of cfDNA from the sample.

Achieving good sensitivity is very important as the concentration of circulating free DNA released by tumour cells is usually in proportion to the stage of cancer [48]. We were able to detect as low as 4.4 mutated copies per ng of DNA against a genomic DNA background also containing the wild type at levels of 565 copies/ng of DNA. We saw assay signal increases of as much as 300% as shown in Figure 5 with these quantities of mutated and wild type DNA. The specificity and sensitivity results are not complete and cannot be fully stated but the data presented and discussed here show that we can get appreciable changes in the electrochemical signal from relatively low copy numbers of the mutated gene compared to the highly abundant wild type gene also present in the sample. Further work will involve fully defining the assay sensitivity and specificity. As circulating nucleic acids are present in blood at ng/mL levels, which based on the fragment length is analogous to a picomolar concentration, a minimum of femtomolar sensitivity will be beneficial for detection of tumour-specific sequences [17]. Many published biosensor studies realised such sensitivity levels through the use of exotic electrode modifications, typically involving the fabrication of electrodes modified with graphene, nanoparticles, carbon nanotubes, etc. In our case, we opted to keep the electrode substrate low cost, easy to produce and coupled to a PCR reaction to achieve the desired sensitivity and specificity. Whilst our approach leads to a trade-off in terms of time to result, it establishes specific amplification and sensitive and specific hybridisation signals, giving confidence in the result whilst achieving an overall time to result which is a significant improvement over the current clinical practice. The ctDNA concentration response shown in Figure 6 shows a clear dose–response effect which predicts that an increase in ctDNA, per unit concentration, will result in a larger electrochemical signal response in the positive direction (i.e., increasing DPV peak current). Since levels of ctDNA are strongly correlated with tumour stage and response to therapy [49], there is a clear potential for this system to be applied in measuring how a patient’s cancer treatment is progressing.

The findings of this study on ctDNA amplification are in agreement with several previous studies that were also able to successfully detect ctDNA KRAS mutations in patient samples using the ddPCR technique [50,51]. Electrochemical detection will quickly and accurately screen for cancer so treatment can be initiated as quickly as possible. Compared to other low-cost mutation detection technique like StripAssay, our sensor device is more reproducible, sensitive, and easier to manufacture and operate, especially from a clinical point of view. In addition, this study shows that electrochemical sensors can be directly coupled to a PCR reaction that uses standard primers and reagents and does not require optimisation, meaning that amplification reactions for other ctDNA markers can be developed off-chip and transferred directly into the assay to produce a ctDNA panel.

The current time to result for cancer detection in a clinical setting is two–three days (including sample transportation) for a non-complicated biopsy analysis and 7–10 days for a complicated biopsy analysis [52]. In the UK, the National Health Service mutation typing following biopsy can take up to nine weeks [52]. In summary, the DNA isolation from blood, clean up and PCR amplification took around 150 min. The ctDNA target incubation took approximately 60 min, while the CV and DPV pre- and post-hybridisation measurements for each electrode took less than 10 min. This gives a sum total of 3.5 h. However, through optimisation and device integration, we believe there is considerable room for optimisation in terms of time to result. The current analysis time of 3.5 h is a big stride towards PoC provision for ctDNA profiling in a healthcare setting. Further optimisation can be made using isothermal amplification which can cut down the number of thermal cycles and, in turn, decrease the overall amplification time from 1 h to 30 min [53]. As our previously published work shows that we were able to detect KRAS amplicons in plasma to mimic a ‘clinical sample’ [54], near future work will explore the detection of non-specified clinical samples containing different KRAS mutations and mutations in other genes involved in cancer, e.g., P53 and BRCA1. Analysing multiple mutations simultaneously in a given sample without prior knowledge of the alterations using multiplex techniques and direct detection of ctDNA from cancer patient samples will support the future direction of PoC clinical testing.

## 4. Conclusions

We were able to successfully produce a simple DNA sensor requiring no labelling processes or external indicators using a multi-carbon electrode. An electrochemical detection scheme involving a DNA hybridisation technique and screen-printed carbon electrodes were developed and shown through a series of comparative measurements to be sensitive and specific for the KRAS G12D and G13D mutations. The DNA modified sensors demonstrated superior performance to electrochemical pre-treatment with acetate buffer containing NaCl and characterisation using ferri-ferrocyanide buffer. Improved sensor sensitivity was achieved by designing a PCR reaction capable of amplifying either mutant KRAS G13D or wild-type KRAS through primer choice from representative patient samples. Cyclic voltammetry and Square Wave measurements were very sensitive for charactering the surface of the modified SPCEs and Differential Pulse voltammetry measurements provided the desired response and indicated detection was possible from samples containing as few as 0.58 ng/µL concentrations of amplicons. In addition, the response was found to be consistent with previously observed results, i.e., large signal decreases being evident upon amplification of the mutant allele, offering the promise of quantitation of mutant sequences from clinical samples. Both non-complementary DNA probes and wild-type DNA amplification reaction was successfully used as control. These results increase the prospect of simple, rapid and cost-effective measurement of nucleic acid tumour markers from blood and other body fluids. The current time to result of the electrochemical sensor was 3.5 h, providing notable scope for optimisation. It is essential to note that the sensor being developed can be potentially used for both early detection of cancer and monitoring the response to cancer treatment.

## Figures and Tables

**Table 1:** List of DNA sequences employed in this study.

**KRAS G13D Probe and Primer Sequences**	
23 Bases Wild-Type Hybridisation Probe	TGGAGCTGGTGGCGTAGGCAAGA
23 Bases Mutant Hybridisation Probe	TGGAGCTGGTGACGTAGGCAAGA
Forward Primer (Wild-Type)	TGTGGTAGTTGGAGCTGGTG
Forward Primer (Mutant)	TGTGGTAGTTGGAGCTGATG
PCR Probe (Mutant)	TCTTGCCTACGCCACCAGCTCCA
Reverse Primer	TTGTGGACGAATATGATCCAACA
**KRAS G12D Probe and Primer Sequences**	
23 bases Wild-type Hybridisation Probe	AGTTGGAGCTGGTGGCGTAGGCA
23 bases Mutant Hybridisation Probe	AGTTGGAGCTGATGGCGTAGGCA
Forward Primer (Wild-type)	TGTGGTAGTTGGAGCTGGTG
Forward Primer (Mutant)	TGTGGTAGTTGGAGCTGATG
Reverse Primer	TTGTGGACGAATATGATCCAACA

## Data Availability

Data is contained within this article and additional data can be found in preceding article https://doi.org/10.3390/IECB2020-07067.
